# Role of Exosomes in Epithelial–Mesenchymal Transition

**DOI:** 10.1021/acsabm.3c00941

**Published:** 2023-12-18

**Authors:** Bikramjit Bhattacharya, Sagnik Nag, Sayantanee Mukherjee, Mrunal Kulkarni, Priti Chandane, Debashmita Mandal, Nobendu Mukerjee, Divya Mirgh, Krishnan Anand, Manab Deb Adhikari, Sukhamoy Gorai, Nanasaheb Thorat

**Affiliations:** † Department of Applied Microbiology, School of Biosciences and Technology, Vellore Institute of Technology (VIT), Vellore, Tamil Nadu 632014, India; ‡ Department of Bio-Sciences, School of Bio-Sciences & Technology, Vellore Institute of Technology (VIT), Tiruvalam Road, Vellore, Tamil Nadu 632014, India; § Amrita School of NanoSciences and Molecular Medicine, Amrita Institute of Medical Sciences, Kochi, Kerala 682041, India; ∥ Department of Pharmacy, BITS Pilani, Pilani, Rajasthan 333031, India; ⊥ Department of Biochemistry, University of Hyderabad, Hyderabad, Telangana 500046, India; # Department of Biotechnology, Maulana Abul Kalam Azad University of Technology (MAKAUT), Haringhata, Nadia, West Bengal 741249, India; ∇ Center for Global Health Research, Saveetha Medical College and Hospital, Saveetha Institute of Medical and Technical Sciences, Chennai, Tamil Nadu 600077, India; ◊ Department of Health Sciences, Novel Global Community and Educational Foundation, Hebersham, New South Wales 2770, Australia; ⧫ Vaccine and Immunotherapy Canter, Massachusetts General Hospital, Boston, Massachusetts 02114, United States; ● Department of Chemical Pathology, School of Pathology, Faculty of Health Sciences, University of the Free State, Bloemfontein 9300, South Africa; ▲ Department of Biotechnology, University of North Bengal Raja Rammohunpur, Darjeeling, West Bengal 734013, India; ▷ Rush University Medical Center, 1620 W. Harrison St., Chicago, Illinois 60612, United States; ◨ Limerick Digital Cancer Research Centre and Department of Physics, Bernal Institute, University of Limerick, Limerick V94T9PX, Ireland

**Keywords:** Exosome, Cancer, Metastasis, EMT, Biomarkers, Therapeutic

## Abstract

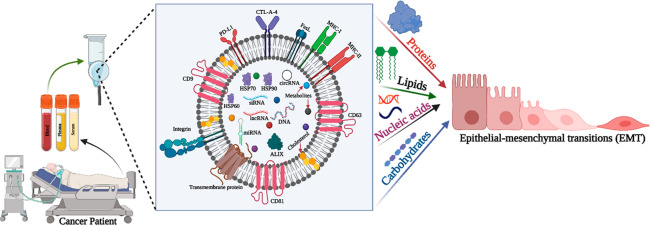

Epithelial–mesenchymal transition (EMT) is a fundamental process driving cancer metastasis, transforming non-motile cells into a motile population that migrates to distant organs and forms secondary tumors. In recent years, cancer research has revealed a strong connection between exosomes and the EMT. Exosomes, a subpopulation of extracellular vesicles, facilitate cellular communication and dynamically regulate various aspects of cancer metastasis, including immune cell suppression, extracellular matrix remodeling, metastasis initiation, EMT initiation, and organ-specific metastasis. Tumor-derived exosomes (TEXs) and their molecular cargo, comprising proteins, lipids, nucleic acids, and carbohydrates, are essential components that promote EMT in cancer. TEXs miRNAs play a crucial role in reprogramming the tumor microenvironment, while TEX surface integrins contribute to organ-specific metastasis. Exosome-based cancer metastasis research offers a deeper understanding about cancer and an effective theranostic platform development. Additionally, various therapeutic sources of exosomes are paving the way for innovative cancer treatment development. In this Review, we spotlight the role of exosomes in EMT and their theranostic impact, aiming to inspire cancer researchers worldwide to explore this fascinating field in more innovative ways.

## 1 Introduction

Cancer, the deadliest noncommunicable disease, arises from uncontrolled cell proliferation and constitutes a significant global health burden. The burden of cancer is expected to grow over the next two decades.^1^ Risk factors such as tobacco use, alcohol consumption, poor nutrition, physical inactivity, and air pollution contribute to cancer and other noncommunicable diseases. Certain chronic infections can also pose risks, particularly affecting low- and middle-income countries.^2^ Cancer is projected to claim nearly 10 million lives globally in 2020, making it the leading cause of death.^3^ Recent cancer research has uncovered intriguing links between cancer and extracellular vehicles (EVs).^4^ All active cells secrete EVs,^5^ which are classified into major subpopulations, including macrovesicles, exosomes, large endosomes, and apoptotic bodies.^6^ Exosomes have emerged as the most prominent EV subpopulation in cancer research, playing a crucial role in cell-to-cell communication. Tumor-derived exosomes (TEXs) and their molecular cargo play a significant part in cancer development and progression.^7−11^ Cancer metastasis, the most complex event in cancer progression, is driven by the core process of epithelial–mesenchymal transition (EMT). During EMT, cells become motile, enter the circulatory system, and develop the ability to form secondary tumors.^12^ TEXs molecular cargos, including proteins, lipids, miRNAs, and surface molecules, promote EMT in cancer.^13^ Circulating exosomes within the human body serve as a valuable source of diagnostic and prognostic markers for cancer. Various exosome sources (mesenchymal stem cell-derived exosomes, immune cell-derived exosomes, plant-derived exosomes,^14^ etc.) exhibit cancer-fighting properties. As a result, exosome-related cancer research has helped unravel many complex aspects of cancer in greater detail. In this Review, we spotlight exosome regulatory activity in EMT and its theranostic applications in cancer treatment.

## 2 Biogenesis and Components of Exosomes

**Figure 1:**
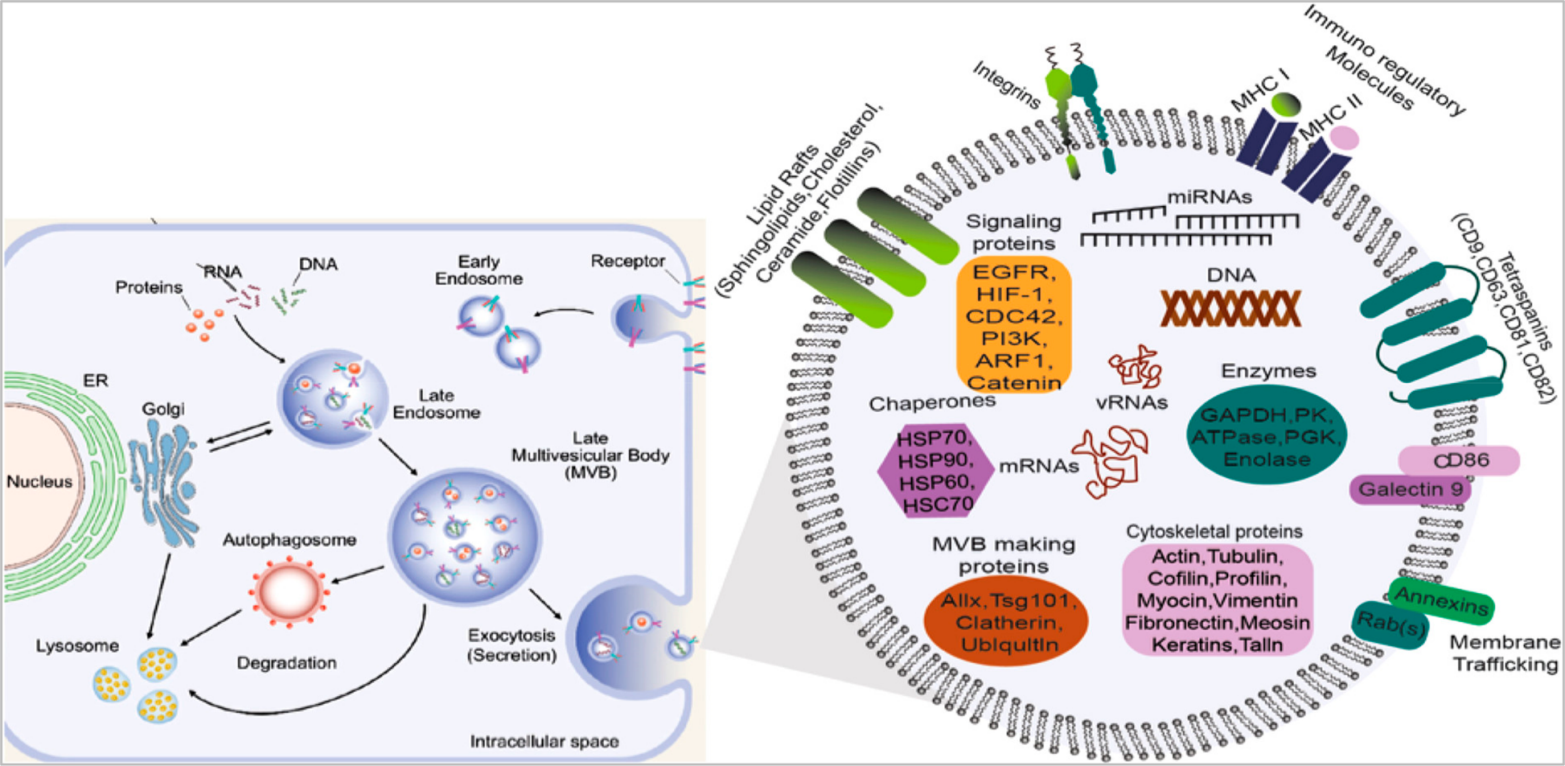
Exosome biogenesis and its molecular cargo (Adapted from ref (106). Copyright 2021 American Chemical Society.).

Extracellular vesicles (EVs) are membrane-bound structures that are found in human blood, plasma, serum, etc.^15^ EV membranes are composed of lipids that resemble the ones present in the plasma membrane of the cell.^16^ It has been established that a large variety of proteins are also integrated into, bound to, or present in the intraluminal area of EVs.^17^ Exosomes are known to be a subset of extracellular vesicles.^18^ It was earlier considered that they carried a cargo of garbage outside the cell,^19^ but in due course, these nanosized vesicles have grabbed great attention among scientists due to their role in cellular communication and cell signaling.^20^ The origin of exosomes from the endosomes (Figure 1) and the intermediate stage is the multivesicular body (MVB) maturing into late endosomes. MVBs carry several intraluminal vesicles (ILVs).^21^ According to recent research, the endosomal sorting complex required for transport (ESCRT) is involved in ILV development. ESCRT has four subsets such as ESCRT-0, ESCRT-I, ESCRT-II, and ESCRT-III. Together, they work in MVB development, cargo selection, and promoting vesicle budding. ESCRT-0 activated ESCRT-I and ESCRT-II. ESCRT-I and ESCRT-II play a role in cargo packaging, and ESCRT-III is involved in vesicle budding.^22^ ESCRT-independent pathway of exosome biogenesis regulated via surface tetraspanin protein, lipids, and ceramide. The composition of exosomes is a combination of many proteins, including receptors, extracellular matrix proteins, transcription factors, enzymes, lipids, and nucleic acids (DNA, miRNA, mRNA, etc.).^13,23^

## 3 Exosomes in Immune Cell Reprogramming and Cancer Progression

**Figure 2:**
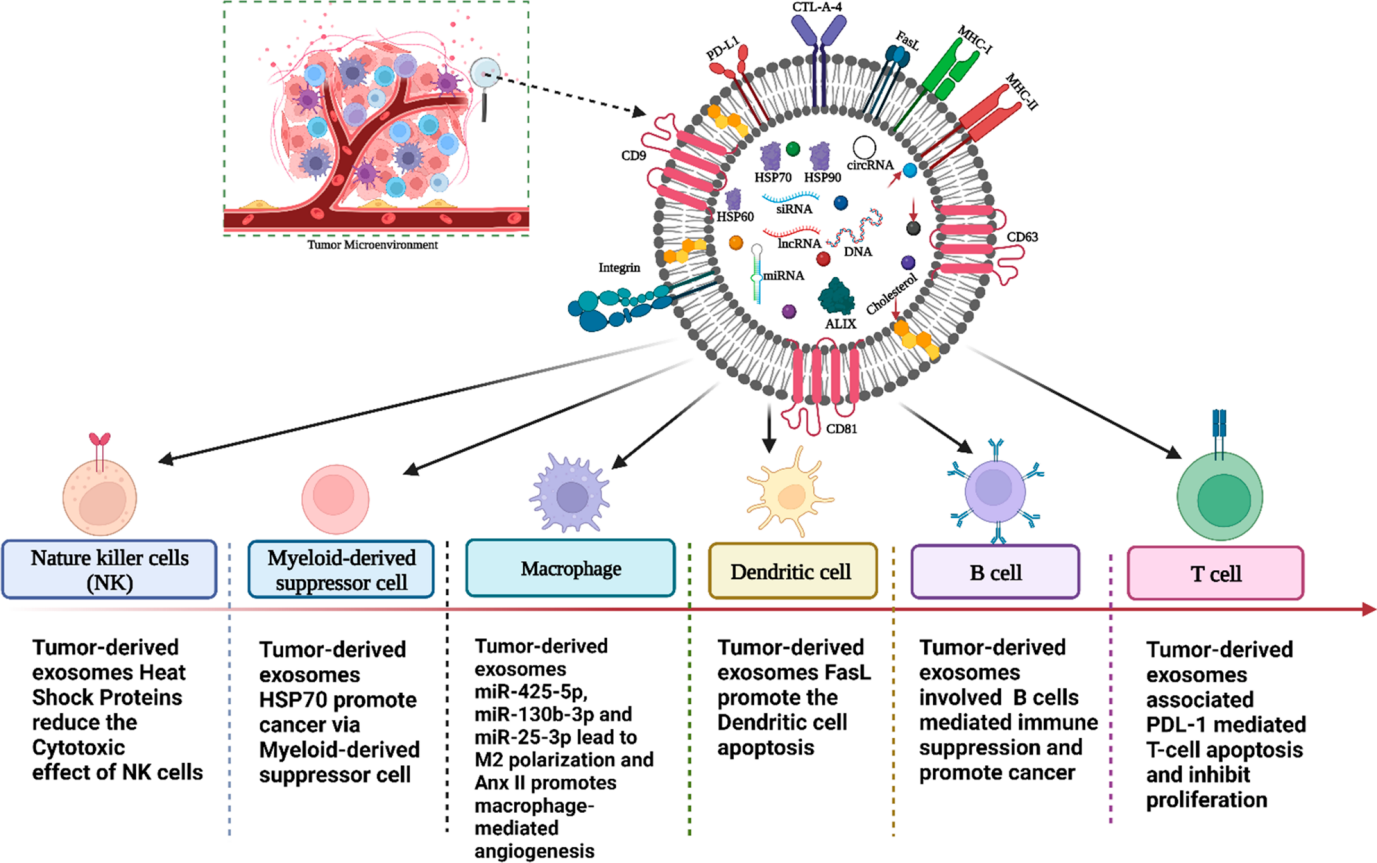
Tumor-derived exosomes (TEXs) alter immune cell function in cancer (created with BioRender.com).

During cancer, immune cell reprogramming is a vital cell event, as a result cancer cells escape the immune surveillance.^121^ TEXs play a major role in the process. They originate from the plasma membrane and dynamic functional biomolecules that signal the target cell to either suppress or stimulate an immune signal in pathological conditions such as infection and cancer.^24^ There has been increasing evidence of how these cargos contained within exosomes modulate myeloid as well as lymphoid cell function.^25^ Myeloid-derived suppressor cells (MDSCs) expand during infection, inflammation, and cancer.^26^ Scientific research evidence suggests that exosomes released by hypoxia-induced glioma cells delivered miRNA-10a and miRNA-21 which promoted MDSC activation and differentiation.^27^ Similarly, exosomes carrying miRNA-9 and miRNA-181a shed from breast cancer cells and miRNA-107 from gastric cancer cells caused the expansion of MDSCs.^25^ Various studies have confirmed that exosomes derived from several cancers promote M1 to tumor-promoting M2 phenotype;^28^ for example, exosomes derived from HCC harboring miRNA-146-5p induced M2 polarization while inhibiting interferon α, β expression, and high expression of program cell death legend-1 reprogramming of T cells mediated an immune response in cancer.^27^ Similarly, the exosome-mediated delivery of miRNA-222 in EOC cells, miRNA-301a-3 in pancreatic cancer cells, and miRNA-425-5p, miRNA-25-3p, and miRNA-130b-3p in colon cancer cells led to the formation of M2 phenotype which promoted tumor growth, EMT induction, and angiogenesis that ultimately enhanced metastasis.^25^ Reprogramming of macrophages through exosome-mediated delivery of miRNA-1246 in p53 mutant cancer cells induced the anti-inflammatory state required for tumor progression.^25^ In another study, exosomes shed from cells of hepatocellular carcinoma under endoplasmic reticulum stress stimulated macrophages to secrete MCP-1, IL-6, IL-10, and TNF-α, whereas cells of breast cancer under ER stress released exosome miRNA-27a-3p upregulated the PD-L1 expression in macrophages leading to immune evasion.^29^ Studies have also indicated that tumor-derived exosomes simulate NF-κB signaling in macrophages.^30^ In gastric carcinoma caused by the Epstein–Barr virus, the exosomes shed by infected epithelial cells inhibited dendritic cell (DC) maturation.^27^ In another study, it was reported that exosomes bearing miRNA-212-3p released by pancreatic tumors caused immune tolerance by inhibiting RFXAP expression and downregulating expression of MHC-II on dendritic cells.^2^ Furthermore, exosomes carrying miRNA-203 in pancreatic cancer cells decreased the TLR-4 expression and interleukin-12 tumor necrosis factor-α in DC. Another study showed that cancer cell-derived exosomes from B-cell chronic lymphocytic leukemia and 4T1 breast cancer suppressed differentiation while inducing programmed cell death and enhancing PD-L1 expression in DCs.^25^ Additionally, T regulatory (Treg) cells have also been shown to modulate DC function via exosome miRNA-142-3p and miRNA-150-5p resulting in tolerogenic DC phenotype.^27^ Studies show that IL-2 inhibits the activation of Natural Killer (NK) cells through exosomes derived from tumor cells. In blood cancer B cells, T cells, and NK cells, immunosuppressive effects are regulated via exosomes. In yet another study, exosomes harboring TGF-β1 released by pancreatic adenocarcinoma cells dysregulated NK cell function by inhibiting TNF-α, INF-γ, NKG2D, and CD107a expression.^27^ In contrast, exosomes bearing HSP70 stimulate the production of INF-γ by NK cells in multiple myeloma cells via activating the NF-κB pathway conferring antitumor immunity. Pancreatic and colon cancer cell-derived exosomes carrying HSP70/Bag4 enhanced migratory potential and stimulated cytotoxicity in NK cells.^27^ TEX’s molecular cargo led to the induction of apoptosis or activation or suppression of T cell function. For instance, exosomes bearing FasL in oral squamous cell carcinoma cells induce apoptosis of T cells via extrinsic and intrinsic pathways.^31^ Similarly, exosomes carrying FasL in prostatic cancer cells also induced programmed cell death in CD8+ T cells and suppressed their growth. FasL-mediated cell death is caused by melanosomes.^32^ Exosome-based skin cancer progression led via INF-γ and PDL-1 to higher expression associated cytotoxic T cell downregulation.^32^ In breast cancer, PDL1-mediated immune suppression takes place in the tumor microenvironment (TME).^27^ Exosomes harboring 14-3-3(phospho-serine binding proteins) released by hepatocellular carcinoma cells inhibited the antitumor effects of T cells in the TME.^27^ In colorectal cancer stem cells, exosome-mediated transfer of miRNA-146a-5p enhanced tumor progression along with the decrease in the number of cytotoxic T cells infiltrating the TME. Exosomes also alter B cell function. HCC cell-derived exosomes are involved in B-cell proliferation, expressing interleukin-10 and inhibiting the proliferation of CD8+ T cells, thereby drastically decreasing the antitumor immunity in TME.^33^ All immune cell alteration development is a favorable condition for cancer progression (Figure 2).

## 4 Exosomes in Extracellular Matrix (ECM) Remodeling in Cancer

**Figure 3:**
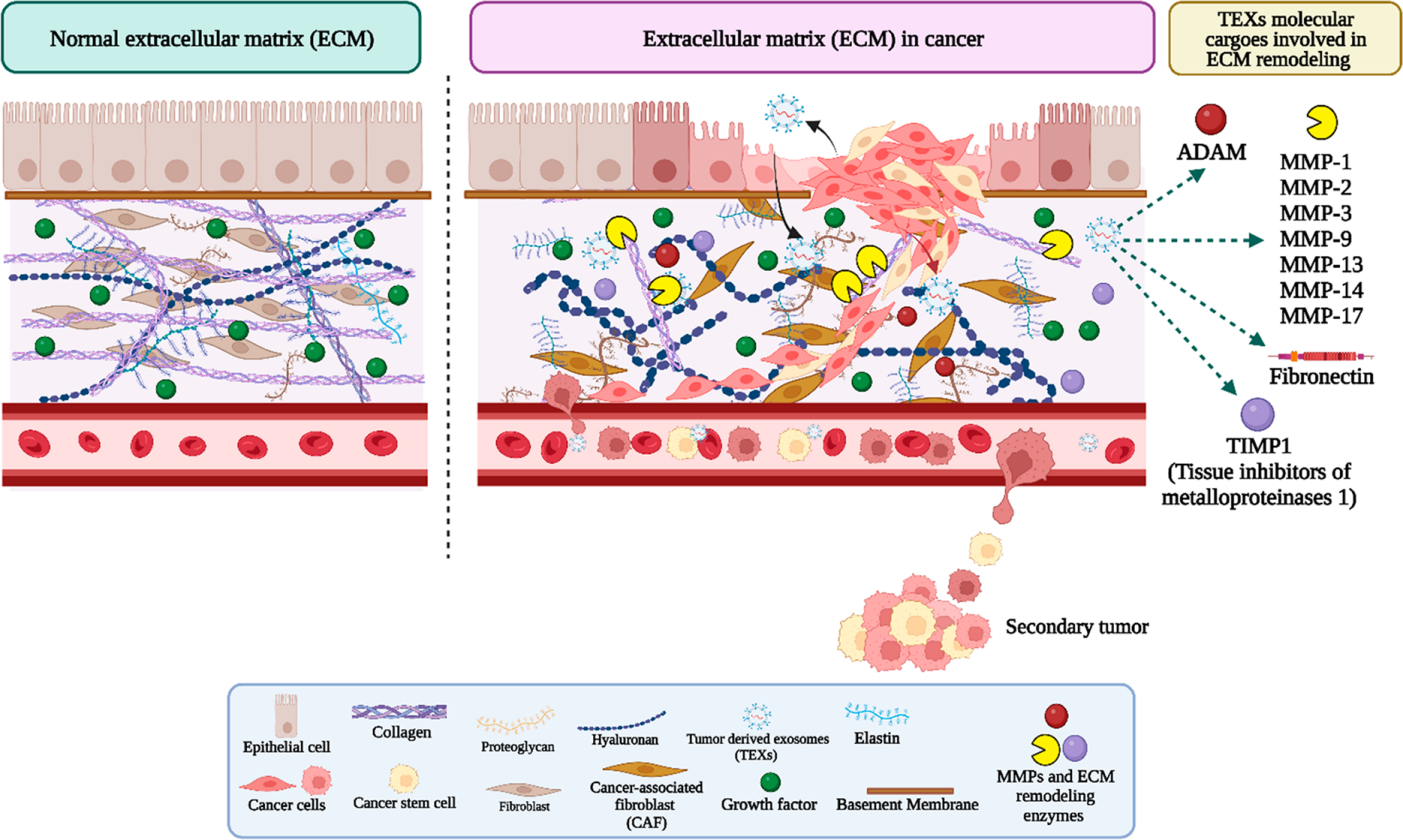
Exosomes in the extracellular matrix remodeling in cancer (created with BioRender.com).

In the tumor microenvironment, cancer cell extracellular matrix remodeling promotes cell motility development. This event leads to metastasis. ECM is composed of protein, glycoprotein, and peptidoglycan. The deep exploration of the exosome and cancer interlink defines tumor-derived exosome (TEX) metalloproteinase (MMPs),^107−115^ ADAM,^116^ and fibronectin involved in ECM^34^ (Figure 3). The protein profiling of ECM events suggests that integrin, annexins, integrin α3, and metalloproteinase participate in cancer cell migration and ECM.^35^ The tumor microenvironment (TME) associated fibroblast is the most influential cell population in cancer development.^36^ This has a major contribution to cancer-associated fibroblast (CAF) development and CAF participation in EMT. Tumor growth and development are regulated via CAF cells secreted by inflammatory signaling molecules and growth factors. Adipocytes from cancer patients have higher levels of IL6, IL1, and MMP11 (CAAs). Adipocytes develop fibroblasts involved in extracellular matrix remodeling, and they support metastasis.^37^ The clinical investigation suggests that in brain cancer the patient’s serum containing the miRNA molecular signature of exosome promotes brain cancer progression. Advanced stage cancer patients carry a huge number of exosomes in their body fluids, and these exosomes develop complex cell signaling toward cancer development.^38^ Exosome long noncoding RNA SNHG3 acted as a sponge for miRNA-330, favorably regulating pyruvate kinase M1/M2 (PKM), decreasing oxidative phosphorylation, increasing glycolysis, and stimulating the proliferation of breast cancer cells.^39^ Exosomes formed from breast-cancer-derived miRNA-105 can alter the metabolism of CAFs. CAFs also had altered metabolic profiles, which promote the growth of cancer cells. Exosomes harboring virus-encoded microRNAs were absorbed by neighboring cells, shifting their metabolism toward glycolysis and limiting mitochondrial biogenesis. Exosomes promoted angiogenesis in Kaposi’s sarcoma by altering the metabolism of neighboring cells. Exosomes deliver angiogenic medicines or microRNA to ECs, altering their metabolism and angiogenic action. Exosomes produced from SMAD4-deficient pancreatic ductal adenocarcinoma (PDAC) cells can induce immunosuppression.^40^ Immunosuppression caused by exosome-mediated metabolic reprogramming may hasten tumor growth. Exosome proteins, microRNAs, noncoding RNAs, and metabolites all have an impact on metabolic reprogramming. In addition to microRNAs, lncRNAs and circRNAs have been studied.^41^ Exosome-based ECM investigation supports the understanding of several facts about cancer metastasis.^10,11,117^

## 5 Role of Exosomes in Cancer Metastasis

**Figure 4:**
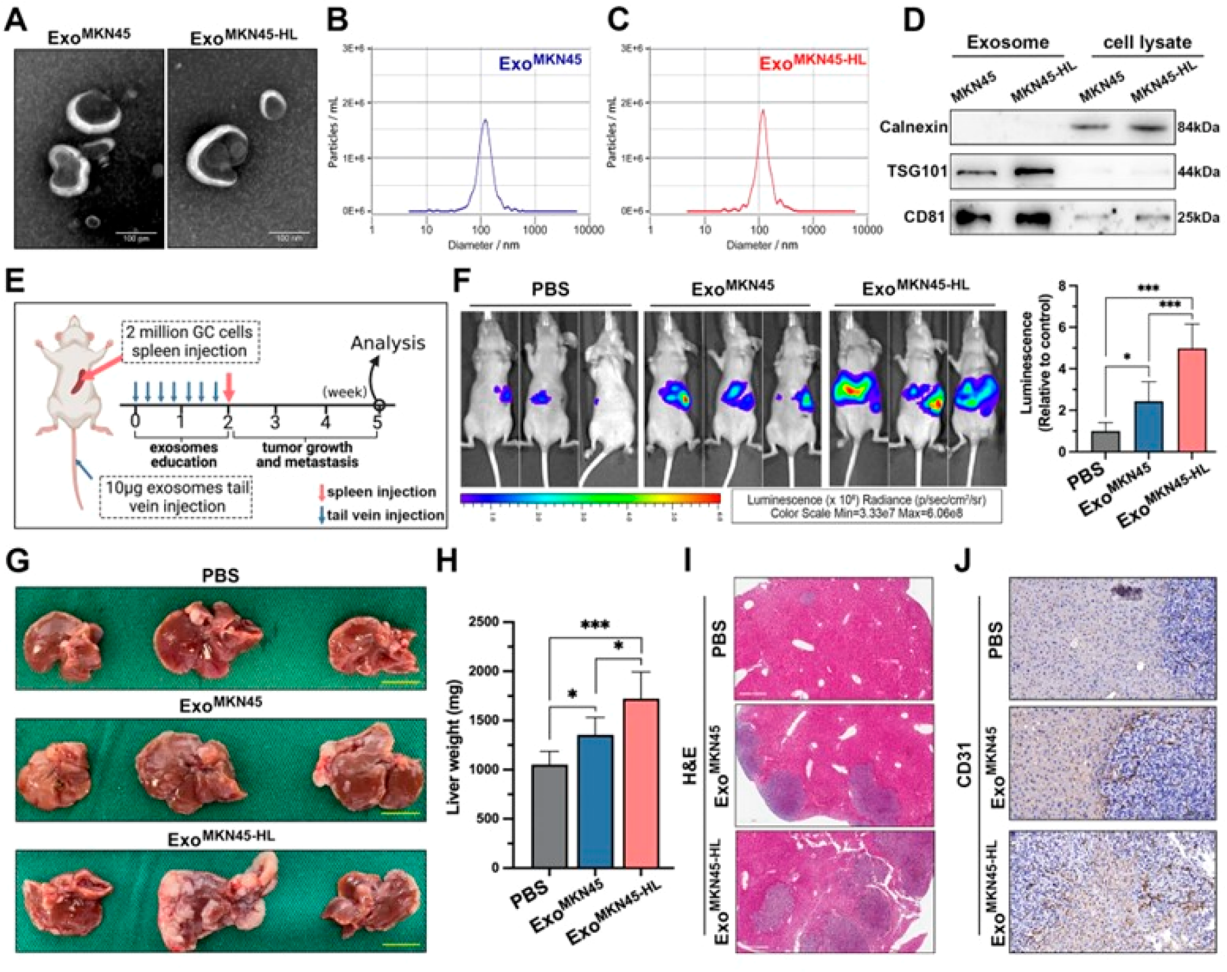
Exosomes associated with liver metastasis in gastric cancer (GC) show distinct characteristics. (A) Transmission electron microscopy (TEM) image of exosomes derived from MKN45 and MKN45-HL cells and (B, C) size distribution analysis of purified exosomes from MKN45 and MKN45-HL cells using NanoSight. (D) Western blot assessment of exosome markers (TSG101 and CD81) in exosomes and lysates from MKN45 and MKN45-HL cells. (E) Diagram illustrating the process of establishing the exosome-informed GC-LM model. (F) Representative in vivo imaging system (IVIS) outcomes in mice injected with luciferase-tagged MKN45 cells into the spleen after being exposed to PBS, MKN45 exosomes, or MKN45-HL exosomes. (G–I) Impact of GC-derived exosomes on liver metastasis in mice, featuring images of liver metastasis, liver weight, and H&E staining. (J) Representative CD31 immunohistochemical staining images of liver metastasis tissues from exosome-educated mice (Adapted with permission from ref (118). The copyright is licensed under a Creative Commons Attribution 4.0 International License 2022, *.J Exp. Clin. Cancer Res.*, Springer Nature).

First observed in developmental biology research during the 1970s, EMT plays a vital role in embryo development and organogenesis.^42^ This process of epithelial cells changing into mesenchymal cells is known as epithelial-to-mesenchymal transition (EMT).^152^ Many essential developmental processes, including the gastrulation process and healing of wounds, rely on this mechanism.^153^ The EMT, on the other hand, can play a role in the progression of cancer by enabling cancer cells to detach from tumors and infect adjacent tissues. A complex system of signaling channels regulates the EMT. Several transcription factors, including ZEB1/2, SNAIL, Slug, and Twist, have been identified as crucial regulators of the EMT.^154^ These factors hinder the expression of epithelial markers like E-cadherin while promoting the production and expression of mesenchymal markers like Vimentin and N-cadherin.^155^ The transcription factors stimulate the loss of epithelial cell–cell adhesion and the emergence of mesenchymal features, allowing cells to migrate and invade by modifying the expression of these proteins.^156^ When these so-called transcription factors are activated, epithelial cells undergo alterations such as loss of polarity, enhanced motility, and invasiveness.^157^ During EMT, epithelial cells lose their unique apical-basal polarity, whereas mesenchymal cells are more invasive and can penetrate the basement membrane. PI3K/Akt, TGF-β, Notch, and Ras/MAPK are all signaling pathways that influence the EMT. These pathways can either activate or decrease the production of EMT-inducing transcription factors, resulting in a change in character from an epithelial to a mesenchymal phenotype.^158^ TGF-β signaling, for example, is a powerful inducer of EMT, and its activation is linked to increased metastasis in cancer.^159^ Exosomes are important in cancer because they induce the EMT, which converts noninvasive epithelial tumor cells into invasive mesenchymal-like cells.^160^ Cancer cells can detach from the main tumor, move through the lymphatic system or through the circulation of blood, and form new metastatic colonies in distant organs as a result of this process.^161^ Exosomes long noncoding RNA play a significant role in EMT promoting.^162^ EMT has been linked to increased cell mobility, invasiveness, and metastasis. Exosome-mediated EMT might be averted; exosomal cargo could be modified to cause disruption with EMT signaling systems, and exosomes could be used as diagnostic or prognostic indicators for EMT-driven malignancies. Understanding these pathways has the potential to lead to the development of innovative treatment techniques for preventing metastasis and improving cancer patient outcomes.^163^ EMT is closely associated with tumor formation, invasion, metastasis, and treatment resistance. Certain cells exhibit both epithelial and mesenchymal EMT markers, forming a hybrid population. The bioactive substances within exosomes^43^ exert unique regulatory effects that promote a shift toward a mesenchymal cell population, triggering the onset of EMT. Snail1-expressing fibroblasts are present in exosomes derived from cancer-associated fibroblasts (CAFs), which inhibit E-cadherin expression and induce EMT in A549 lung cancer cells. In bladder cancer, CAF-secreted exosome-mediated IL-6 signaling fosters an aggressive cancer pattern.^44^ In cancer, the fibrosis process is linked with cancer development.^137−139^ This process is continued via chronic inflammation (20% of cancer related to this phenomenon) or viral infection. Extracellular vesicles (EVs) have strong participation in this process.^137^ Tumor-derived exosomes (TEXs) promote cancer metastasis (Figure 4).^118,132−135^

TEXs carry a small protein called MAP17 between tumor cell subsets to enable horizontal spread and EMT. MiRNA-92a-3p is found in significant concentrations in liver and colon cancer.^45^ In liver cancer, PTEN expression is suppressed by exosome miRNA-92a-3p and enhances metastasis in colon cancer. In breast cancer, exosome miRNA-181 enhances metastasis. M2 is the cancer-promoting macrophage and suppresses the immune system.^46^ CAF-derived exosome miRNA-342 plays a role in immune suppression. HOTTIP, an exosome lncRNA, promotes its target HMGA1 in gastric cancer (GC) cells, causing EMT and cisplatin resistance. One of the most exciting facts is that exosomes also play a vital role in radiotherapy resistance.^119^ Exosomes play a vital role in colorectal cancer EMT.^47^ Circular RNA (noncoding RNA), another group of exosome RNA, has strong regulatory activity in cancer metastasis. In prostate cancer, circular RNA and miRNA-582 promote EMT. Circular RNA influences several signaling cascades in lung cancer.^48,49^ The participation of the exosome molecular signature in EMT is explained in Table 1.

**Table 1:** Exosome-Associated Molecular Cargos Interlink in EMT

exosome cargos	molecules	involvement in EMT	references
Proteins	PDL1	Induce the immune cell’s apoptosis and suppress the immune system	(27)
	FasL	Induce cytotoxic T cells apoptosis and suppress the immune system	(31)
	Tetraspanins (CD9, CD63, CD81, CD151)	Cancer angiogenesis, metastasis, EMT	(100)
	CD97	Premetastatic niche in gastric carcinoma cells	(58)
	Annexins, integrin α3, fibronectin, and metalloproteinase	Extracellular matrix remodeling	(34, 35)
	Integrin	Organ-specific metastasis	(67)
	Heat shock proteins	It reduces NK cell-based anticancer response in cancer	(101)
Lipids	Membrane lipid	Cell signaling	(104, 105)
RNA	miRNA-9 and miRNA-181a	Breast cancer progression	(25)
	miRNA-425-5p, miRNA-130b-3p, and miRNA-25-3p	M2 polarization	(25)
DNA	Genomic DNA, mitochondrial DNA, oncogenic DNA	Reflect on the parental cell mutation status and it also related epigenetic regulation	(102, 9)
Carbohydrate	Glycans	Cancer metastasis	(103)

## 6 Role of Exosomes in Premetastatic Niche Formation

**Figure 5:**
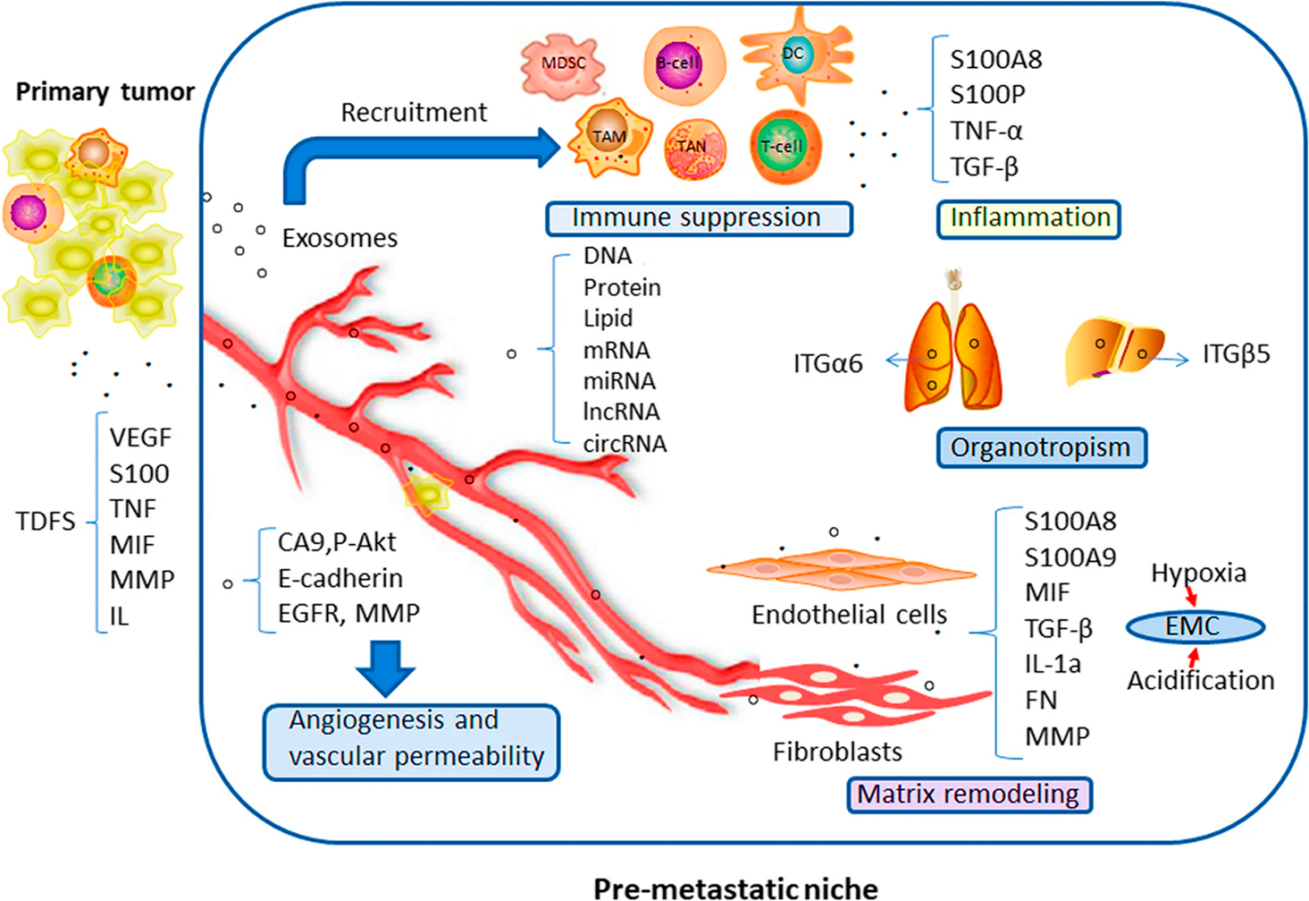
Exosome involvement in premetastatic niche formation (Adapted with permission from ref (120). The copyright is licensed under a Creative Commons Attribution 4.0 International License 2019, Molecular Cancer, Springer Nature).

The formation of a premetastatic niche (PMN) (a tumor-driven environment in a distant organ) supports the growth and survival of metastasized tumor cells. The development of secondary lesions significantly contributes to cancer-related deaths. Over the past decades, research has suggested the potential role of tumor-derived extracellular vesicles in regulating PMN.^50^ Cao^58^ outlined four critical components that drive metastasis niche formation, including bone-originated cells, stromal cells, immune cell suppression, and tumor-derived secreted factors (TDSFs), such as cytokines, growth factors, interleukin-1, tumor necrosis factor-α, β, and vascular endothelial growth factor (VEGF).^51^ These factors converge at premetastatic sites before the accumulation of cancer cells, often in organs distinct from the primary tumor site.^52^ By acting in a paracrine manner on tumor cells, TDSFs promote their migration toward potential PMN formation sites.^53^ TDSFs may activate host stroma within premetastatic niches to induce the expression of pro-inflammatory components. PMNs are regions where immune cells, such as bone marrow-derived cells (BMDCs), are actively recruited, leading to increased secretion of inflammatory components. Inflammatory elements, transported via the bloodstream, eventually reach PMNs on exosomes isolated from the tumor, transforming the PMN into a tumor-supportive, inflamed microenvironment.^54^ Both tumor cells and stroma-derived exosomes enhance systemic infiltration and progression of tumor cells throughout the metastatic cascade,^55,56^ influencing various cancer hallmarks such as uncontrolled cell growth, angiogenesis, and metastasis. Exosomes can directly impact potential metastatic tissue growth and initiate PMN development by altering local factors like cell population, nutrient availability, and vascularization or by influencing the creation of a permissive microenvironment that allows bone marrow-derived cells, like mesenchymal stem cells (MSCs), to migrate to the tumor site and prime the parenchyma for cancerous cells.^57^ This evidence highlights the pivotal role of exosomes in cancer metastasis as they are involved in establishing and maintaining PMNs. Notably, exosome CD97 is linked to the formation of a PMN in gastric carcinoma cells,^58^ while exosome miRNA-21 and miRNA-29a trigger inflammatory responses during this process.^59^ Exosomes also express PD-L1-mediated immune cell evasion, promoting PMN formation (Figure 5).^120^

## 7 Role of Exosome in Organ-Specific Metastasis

**Figure 6:**
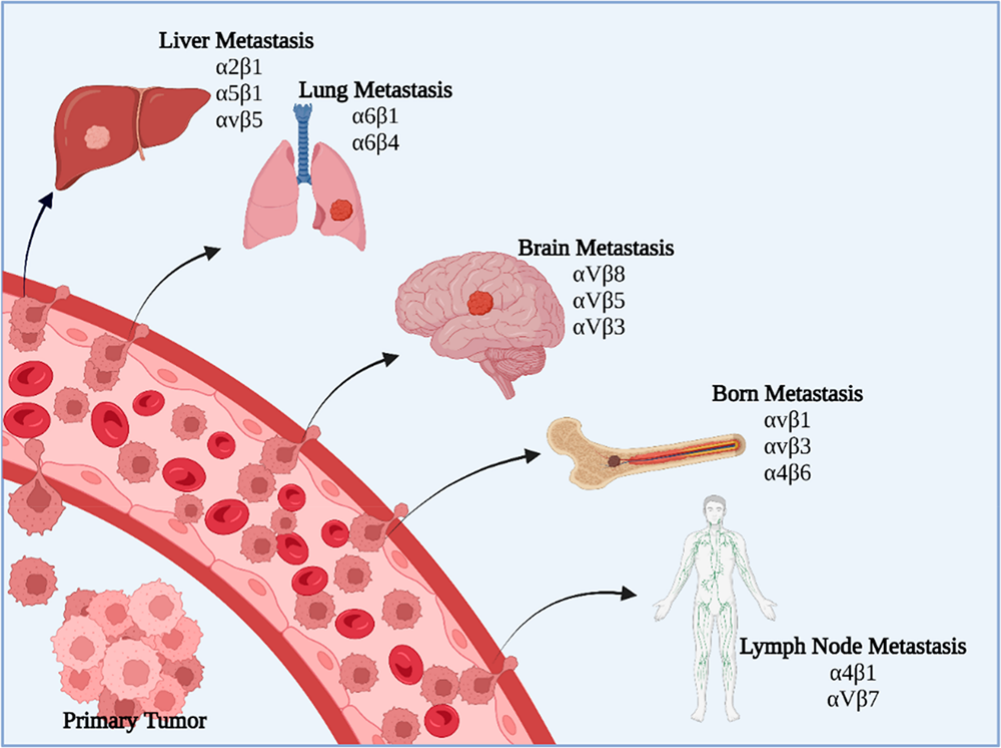
Tumor-derived exosome (TEX) integrins led to organ-specific metastasis (created with BioRender.com).

Metastasis is the most devastating stage in cancer, which when established successfully in a distant vital organ makes the root cause of cancer irreversible to a certain extent. Metastasis is caused when cancer cells migrate to another organ and develop a secondary tumor.^60^ There are not any known mechanisms that link EMT plasticity to organotropism metastasis, but research suggests that epithelial plasticity governs cancer stemness, and cancer stem cells (CSCs) are what cause organotropism metastatic.^61−63^ This entire process of metastasis is carried out on the molecular and cellular levels and even on the genomic level. Recent studies have shown the role of extracellular vesicles in giving guidance to circulating cancer cells to migrate to specific organs and form a secondary tumor. Specifically, extracellular vesicle surface molecules (integrins principle one) promoted metastasis.^64,65^ The role of integrin in recent years has been associated with metastasis due to its property of two-way receptor signaling. It is evident that constitutive activation of integrins by internal stimulation results in better adherence to the ECM and, consequently, a more complex interplay of these adhesion receptors with their substrates. Several worldwide studies recommend that TEX surface integrins are the major ingredient that influences organ-specific metastasis in cancer.^66−68^ Integrins are the glycoprotein that contracts by two subunits, α and β. Based on this subunit, exosomes guide the circulated cancer cell to migrate to different organs and form a secondary tumor such as bone (α4β1, αVβ6, αVβ3), brain (αVβ3, αVβ5, αVβ8), liver (α5β1, α2β1, αVβ5), lymph node (α4β1, αVβ7), and lung (α6β1, α6β4) (Figure 6).^68,69^ Tumor-derived exosomes (TEXs) reprogram the immune system to promote cancer progression.^58,69,70^

## 8 Exosome Source of Cancer EMT Biomarkers

**Figure 7:**
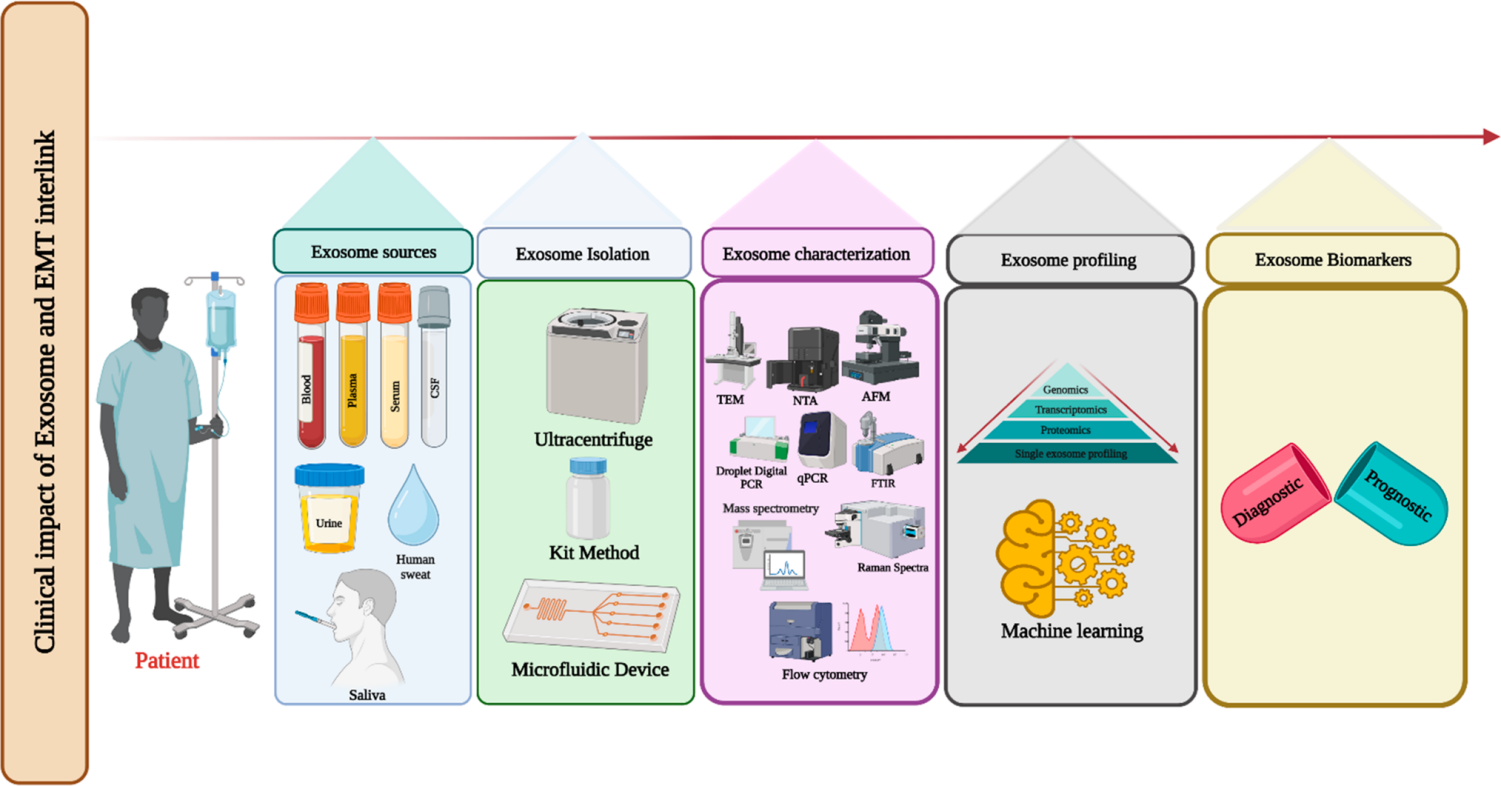
Clinical importance of exosomes and EMT interlink (created with BioRender.com).

Cancer biomarker research is the most exciting event because this process can only give guidance on proper treatment and understanding of the complication level of the disease. TEXs are a promising source of dynamic biomarkers of cancer (Figure 7). Exosome-derived EMT biomarkers have the potential to reveal ground-breaking insights in cancer research, illuminating the intricate mechanisms driving cancer development. These cutting-edge biomarkers provide a glimpse into the mystifying realm of EMT, setting the stage for remarkable breakthroughs in cancer diagnosis, prognosis, and therapy. By tapping into the capabilities of exosome-related EMT biomarkers, we can transform our understanding of cancer and usher in an era of hope and promise for patients across the globe.

With breast cancer topping global cancer statistics as the most prevalent cancer,^71^ cutting-edge diagnostic methods such as exosome-associated CD82 are emerging as valuable biomarkers for early detection.^72^ Exosome research has revealed a treasure trove of essential biomarkers for various cancers, including saliva-derived exosomes in oral cancer^122^ and plasma-derived exosome miRNA-222 as a prognostic marker for breast cancer.^73^ Sweat and tear exosomes are also a source of cancer biomarkers.^131,136^ In lung cancer, serum-derived exosome miRNA-106b^74^ serves as a diagnostic tool, while plasma-derived exosome miRNA-21 and miRNA-4257^75^ provide prognostic insights. For liver cancer, diagnostics and prognostics are enhanced by serum-derived exosome circular RNA^76^ and serum-derived exosome miRNA-1262. Research on colon cancer has uncovered exosome-associated biomarkers CD147 (blood-based diagnostic marker)^77^ and miRNA-486-5p (prognostic marker).^78^ Diagnostics and prognostics for brain cancer have been advanced by serum-derived exosome miRNA-182-5p^79^ and miRNA-301a,^80^ respectively. Table 2 explores EMT-related biomarkers in greater detail, highlighting the incredible potential of exosome research in transforming cancer detection, prognosis, and treatment for the most common cancers worldwide.

**Table 2:** Exosome-Associated Molecular Significance as an EMT Biomarker

exosome-related EMT biomarker	exosome source	molecules	function	references
Diagnostic marker	Blood	CD82	Breast metastasis	(72)
	Serum	miRNA-106b	It associated with lung lymph node metastasis	(74)
	Serum	circRNA-100	Liver cancer tumor metastasis	(76)
	Blood	CD147	Its high expression in advanced stage colon cancer	(77)
	Serum	miRNA-182-5p	High expression in brain cancer	(79)
Prognostic marker	Plasma	miR-222	High expression led to NF-κB mediated breast cancer lymphatic metastasis	(73)
	Plasma	miR-21	Lung cancer	(75)
		miRNA-4257		
	Serum	miRNA-1262	Lower expression in liver cancer	(102)
	Plasma and Serum	miRNA-486-5p	Colon cancer lymph node metastasis	(78)
	Serum	miRNA-301a	High expression in brain cancer	(80)

## 9 Exosome-Based Therapeutic Approach for the EMT

**Figure 8:**
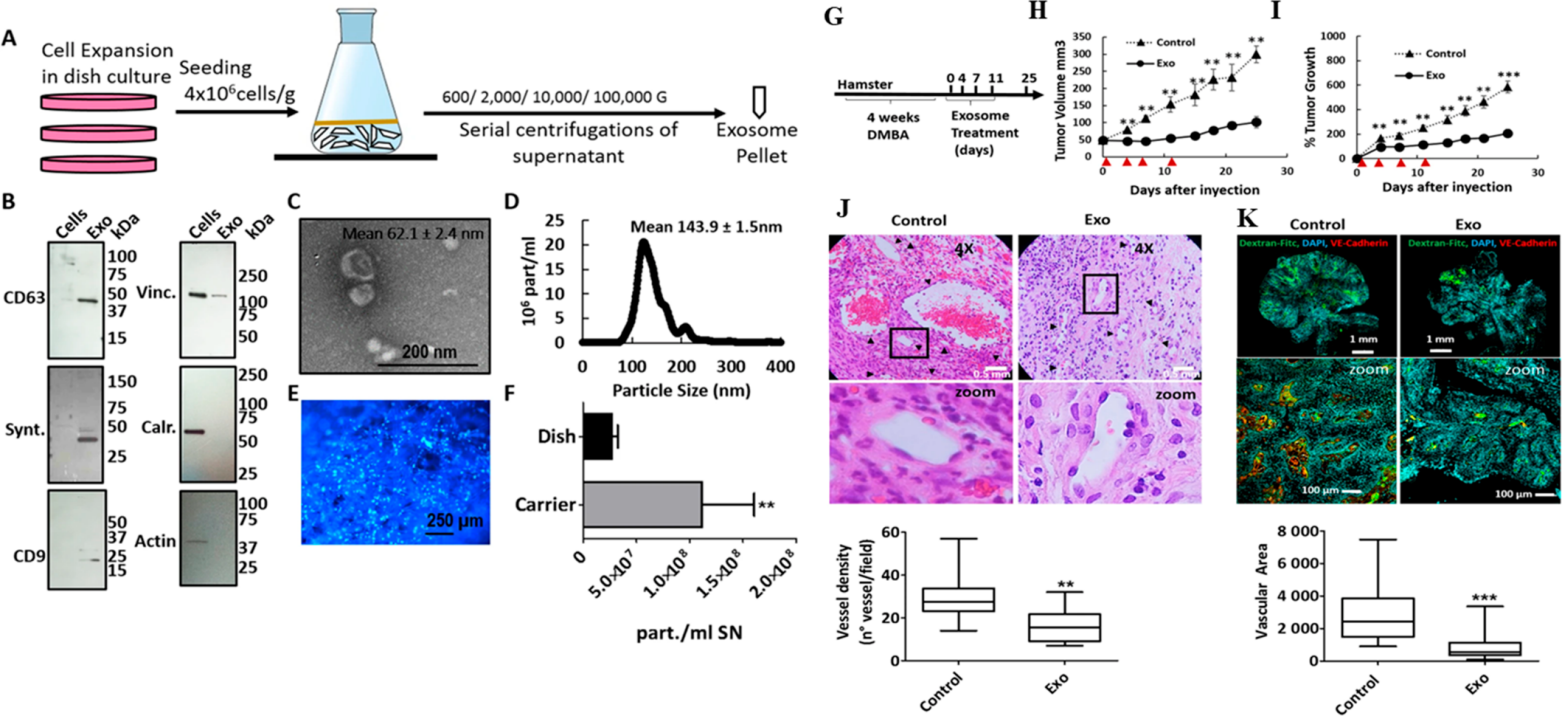
Stem cell derived exosomes inhibit tumor growth and angiogenesis. (A) Exosome isolation protocol, (B) Western blot analysis of exosome biomarkers (negative exosome markers Vinculin (Vinc.), Calreticulin (Calr.), and β-Actin (Actin)). (C) Image of purified exosomes in scanning electron micrograph. (D) Size distribution of exosomes determined by nanosight. (E) Hoechst-stained MenSC on BioNOC II carrier, showing a typical confluence for exosome production. (F) Yield of purified exosomes in PBS as Particles (part)/mL of initial cell culture supernatant (SN) cell lysate (Cells) and exosomes. (G) Experiment plan. Tumors were induced with 4 weeks of DMBA treatment, and four injections of exosomes were administered every 3–4 days. (H, I) Tumor growth in mm^3^ tumor volume and relative tumor growth after days of exosome treatment. Control tumors are shown as triangles and exosome-treated tumors, as circles. (J) Histological sections of tumors at day 25 (end-point) with Hematoxylin and eosin stain (H&E). Quantification of vessel density based on H&E sections is shown on bottom. (K) Dextran-Fitc (green), VE Cadherin (red), and Hoechst (blue) stained histological sections of tumors at day 25 (end-point). (Adapted with permission from ref (124). The copyright is licensed under a Creative Commons Attribution 4.0 International License 2019, Scientific Reports, Springer Nature).

Epithelial to mesenchymal transition is the key basis of metastasis in cancer progression. During the EMT, the cancer cells attain various properties such as self-renewal, resistance against apoptosis, and initializing of the tumor. All of these help the few cancer cells to colonize a distant organ and transform it into a secondary infection site.^81,82^ During the EMT, cancer cells develop radiation resistance and chemoresistance which make cancer treatment more challenging.^83−85^ The ABC transporter protein is the major molecular component that pumps out the drug from the cancer cells, and as a result, it leads to drug resistance in cancer. EMT regulatory several transcription factors alter the apoptosis phenomena in cancer cells.^86,87^ The signaling pathway targeting may be a promising approach to reducing EMT and tumor resistance to therapy. A variety of signaling pathways are involved in the EMT of tumor cells. The EMT is governed by a well-established signaling system known as TGF-/Smad signaling. Gastric cancer and glioma cells have shown that the TGF-receptor inhibitors LV2109761 and LV364947 impede the EMT brought on by ionizing radiation, increasing tumor cells’ irradiation sensitivity.^88,89^ Exosome is a promising approach for cancer prevention (Figure 8). The recent era of treatment focuses mainly on plant-derived exosomes for drug delivery. Future medical applications for drug delivery systems could benefit from the specificity of plant-derived exosomes provided by particular orientations as well as their capacity to transport hydrophobic medicines, modify genes for therapeutic purposes, and avoid immunological rejection.^90^ Plant-derived exosomes (PDXs) are a potential therapeutic tool for cancer with low toxicity.^91^ Mesenchymal stem cell (MSC) derived exosomes show EMT inhibition properties in lung cancer.^92^ Several research investigations show that exosome-based cancer therapeutic development is more promising.^93^ Exosome-based cancer therapy is the beginning of a next generation of cancer treatment.^123^ Although exosome-based cancer therapeutic approaches show promising results, there are some questions that have not been solved such as exosome heterogeneity, large-scale production, diversity of beach production (individual batches get different exosomes), and therapeutic exosome toxicological investigation.^140−143^ Stem cell-derived exosome is a potential source of therapeutic exosomes.^144^ Research evidence indicates that stem cell exosomes also promote cancer;^145^ if stem cell-derived exosomes are not modified, they can promote cancer or inhibit it.^144^ TEXs show anticancer activity,^146^ but their internal oncogenic cargo support plays a dual nature in cancer treatment.^147^ Plant-derived exosomes show effective anticancer activity against cancer, but more toxicological investigation is required.^148^ Toxicological effects of CAR-T cell derived exosomes are low, compared to CAR-T cells.^149^ Artificial chimeric exosomes are another type of exosome that overcomes production limitations with effective anticancer activity with low toxicity.^150^ Immune cell-derived exosomes are also a promising source of cancer therapy.^151^ Exosome-based immunotherapy needs more investigation for standard therapeutic development.^152^ Finally, the exosome-based therapeutic approach needs more time for proper clinical investigation and effective and affordable cancer therapeutic development.

## 10 Future Orientation of Exosome-Based Cancer Research

**Figure 9:**
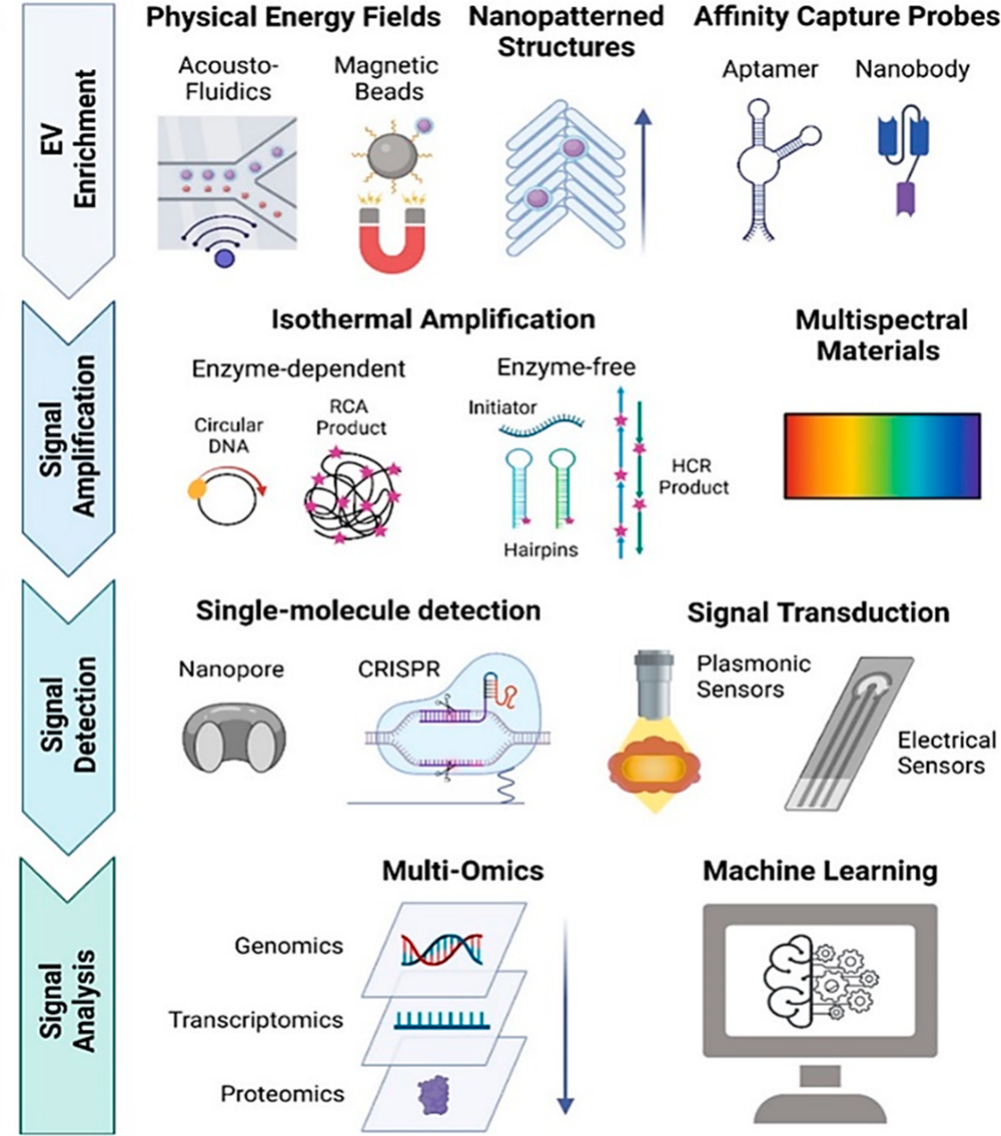
Single exosome profiling (Adapted from ref (126). Copyright 2022 American Chemical Society).

Exosome and cancer association lead to cancer progression and development. It has successfully proven to be a great regulator in the field of oncology due to its dynamic roles in cancer, such as immune cell reprogramming, extracellular matrix remodeling,^125^ premetastatic niche formation,^94^ initiation of metastasis,^95^ and finally organ-specific metastasis,^67^ and most importantly EMT.^96^ The clinical impact of exosomes is significant. Exosomes are adding an impactful aspect to liquid biopsy. Circulated exosome is an emerging source of a cancer biomarker (diagnostic and prognostic).^38^ The therapeutic domain of exosomes has a signature landmark. It is also a potential cancer drug delivery tool. The engineered exosomes^97^ are showing more effectiveness in therapeutic applications. Exosome research faces some questions such as heterogeneity,^126−128^ isolation of golden standard protocol, etc.^98^ The single exosome profiling method decodes this complication (Figure 9). In recent times, muti-omics and machine learning have also come into exosome research.^126^ The intradisciplinary research approach in exosomes supports the development of efficient and affordable solutions for cancer.^129^ The worldwide large-scale scientific mind works on its limitations. The exosome is the brightest star in future cancer precision medicine.^99,130^

## 11 Conclusion

Exosome-based cancer research not only is an innovative approach but also holds the key to unlocking the intricate enigma of the EMT in cancer. Tumor-derived exosomes (TEXs) have been instrumental in deciphering the complex concepts and mechanisms underlying cancer progression. Moreover, exosomes are emerging as potent tools in cancer therapy, with applications in drug delivery and harnessing the potential of various therapeutic exosome sources such as stem cell-derived, immune cells derived, chimeric exosomes, modified exosomes and plant-derived exosomes. To fully capitalize on these advancements, we must focus on creating nanotechnology-based smart platforms for efficient exosome isolation and molecular profiling. As we venture into this transformative liquid biopsy era, we edge closer to realizing the dream of precision oncology. By embracing the power of exosomes, we can revolutionize cancer research and treatment, ultimately improving patient outcomes and saving countless lives.
